# Temperature-Independent Gas Pressure Sensor with High Birefringence Photonic Crystal Fiber-Based Reflective Lyot Filter

**DOI:** 10.3390/s19235312

**Published:** 2019-12-02

**Authors:** Bo Huang, Ying Wang, Chun Mao

**Affiliations:** 1 Guangdong and Hong Kong Joint Research Centre for Optical Fibre Sensors, College of Physics and Optoelectronic Engineering, Shenzhen University, Shenzhen 518060, China; ygkrhb@163.com (B.H.); maochun2017@email.szu.edu.cn (C.M.); 2 Guangdong Laboratory of Artificial Intelligence and Digital Economy (SZ), Shenzhen University, Shenzhen 518060, China; 3 Key Laboratory of Optoelectronic Devices and Systems of Ministry of Education and Guangdong Province, Shenzhen University, Shenzhen 518060, China

**Keywords:** optical fiber sensor, polarization interference, gas pressure sensor

## Abstract

A novel temperature-independent gas pressure sensor based on a reflective fiber Lyot filter is presented in this paper. The reflective fiber Lyot filter is simply consist of a fiber polarizer and a segment of hollow-core photonic bandgap fiber (HB-PCF). The HB-PCF plays the role of birefringent cavity in the reflective fiber Lyot filter and works as the sensor head in the gas pressure sensor. Experiment results show that the responses of the sensor to gas pressure and temperature are 3.94 nm/MPa and −0.009 nm/°C, indicating that the proposed gas pressure is sensitive to gas pressure rather than temperature. Coupled with the advantages of simple structure, easy manufacture, high sensitivity and temperature independent, the proposed reflective fiber Lyot filter-based gas pressure sensor holds great potential application in the field of gas pressure monitoring.

## 1. Introduction

Gas pressure, as a physical parameter reflecting the force that the gas applies on the walls of its container, is an important parameter for ensuring the secure and efficient operation of various industrial equipment, including gas turbine, coal boilers, and so on. Hence, the research on the measurement of gas pressure has become a focus of attention in recent years, and a variety of gas pressure sensors have been proposed and developed by researchers in succession. With the characteristics of light weight, high temperature resistance, and anti-electromagnetic interference, fiber-based gas pressure sensors [1,2,3,4,5,6,7,8,9,10,11,12,13,14,15,16] have stood out from the numerous gas pressure sensors and are well suited for gas pressure measurement in various industrial equipment.

Nowadays, according to the working principle, the typical fiber-based gas pressure sensors can be mainly divided into two categories: Optical fiber grating-based gas pressure sensor [1,2,3] and optical fiber interferometer-based gas pressure sensor [4,5,6,7,8,9,10,11,12,13,14,15,16]. For the optical fiber grating-based gas pressure sensor, its sensor head is typically constituted by a fiber grating, such as the long-period grating (LPG) inscribed by periodically inflating photonic crystal fiber (PCF) [1] or hollow-core photonic bandgap fiber (HC-PBF) [2], and the fiber Bragg grating (FBG) written in a bare fiber [3]. However, the maximum gas pressure sensitivity achieved by the abovementioned sensors is only 1.68 nm/MPa [1], which limits their further practical applications. Compared with the optical fiber grating-based gas pressure sensor, the optical fiber interferometer-based gas pressure sensor is more widely used due to its inherent characteristics of simple structure and high sensitivity. So far, the optical fiber interferometers used in the gas pressure sensors mainly contain Mach–Zehnder interferometers (MZI) [4,5,6,7] and Fabry–Perot interferometers (FPI) [8,9,10,11,12,13,14,15,16]. The MZIs constituted by twin-core fiber [4], inner air-cavity in single mode fiber (SMF) [5], dual side-hole fiber (DSHF) [6], and HC-PBF [7] have been applied in the field of gas pressure detection, and yet for the above MZI-based gas pressure sensors [4,5,6,7], it is essential to employ a femtosecond laser to drill micro-channel on the interferometer structure, which sharply increases the cost. In contrast, more research efforts have been carried out in FPI-based gas pressure sensors for their easy fabrication, low cost, and high sensitivity. The FPIs based on hollow-core fiber (HCF) [8,9] or HC-PBF [10] have been proposed and demonstrated for gas pressure sensing. Meanwhile, the diaphragm-based FPI also provides a convenient way to measure the gas pressure. Different materials, including silica [11,12,13], graphene [14], silver [15], and silk fibroin [16], have been used as the diaphragm in the diaphragm-based FPI-based gas pressure sensors. Nevertheless, under the limit of material properties, this kind of gas pressure sensor cannot operate at high gas pressure.

Lyot filter, which is typically fabricated by inserting a birefringent medium between two polarizers, is a kind of polarization interference filter. Since first being reported in 1933, bulk Lyot filter [17,18], bulk-fiber mixed Lyot filter [19,20] and all-fiber Lyot filter [21,22,23,24,25] have been developed in succession and applied in the fields of spectral imaging [17,18], lasers [19,20], and communication [21], respectively. Recently, Lyot filter has also been employed as optical fiber sensor to measure different physical parameters, such as temperature [22], torsion [23,24], and transverse load [25]. Recently, photonic crystal fiber (PCF), as a new kind of optical fiber, has attracted increasing research interest for its potential application in the fields of optical fiber lasers [26,27,28] and fiber sensing [29]. High birefringence can be introduced into the PCF by the asymmetric air/silica microstructure [30]. For conventional birefringence fibers (e.g., PANDA fiber and bow-tie fiber), as they have large thermal expansion coefficients, their birefringence is sensitive to temperature, which has been employed in fiber sensors for highly sensitive temperature sensing. However, for the conventional birefringence fibers-based fiber sensors, when they are applied for other sensing instead of temperature, for instance, gas pressure, the inherently high temperature sensitivity induces serious cross-sensitivity, which severely limits their practical applications in gas pressure measurement. So far, few works about the conventional birefringence fibers-based fiber sensors for gas pressure sensing have been reported. In comparison, the high birefringence photonic crystal fiber (HB-PCF) exhibits an ultra-low thermal expansion coefficient because of its inherent air/silica microstructure cladding, which can reduce the temperature cross-sensitivity effectively [30], so the HB-PCF-based fiber sensor is a good candidate for gas pressure sensing.

In this paper, we propose and demonstrate a temperature-independent gas pressure sensor with high birefringence photonic crystal fiber-based reflective Lyot filter. The proposed reflective Lyot filter is constructed by simply connecting the fiber polarizer to the HB-PCF with a well-cleaved end. By monitoring the wavelength of the interference dip, the HB-PCF-based reflective fiber Lyot filter can achieve the measurement of gas pressure with the sensitivity of 3.94 nm/MPa. To the best of our knowledge, this is the first time that the reflective Lyot filter has been applied in the field of gas pressure sensing. Moreover, benefitting from the ultra-low thermal expansion coefficient of HB-PCF, the HB-PCF-based reflective Lyot filter overcomes the temperature cross-sensitivity faced by conventional birefringence fibers-based fiber sensors. Experimental research shows that the temperature sensitivity of the proposed sensor is low to −0.009 nm/°C, corresponding to the temperature cross-sensitivity of −2.28 × 10^−3^ MPa/°C and indicating that the proposed sensor is temperature independent. All of the above experiment results confirm the good performance of the proposed reflective fiber Lyot filter-based gas pressure sensor.

## 2. Device Fabrication and Working Principle

**Figure 1:**
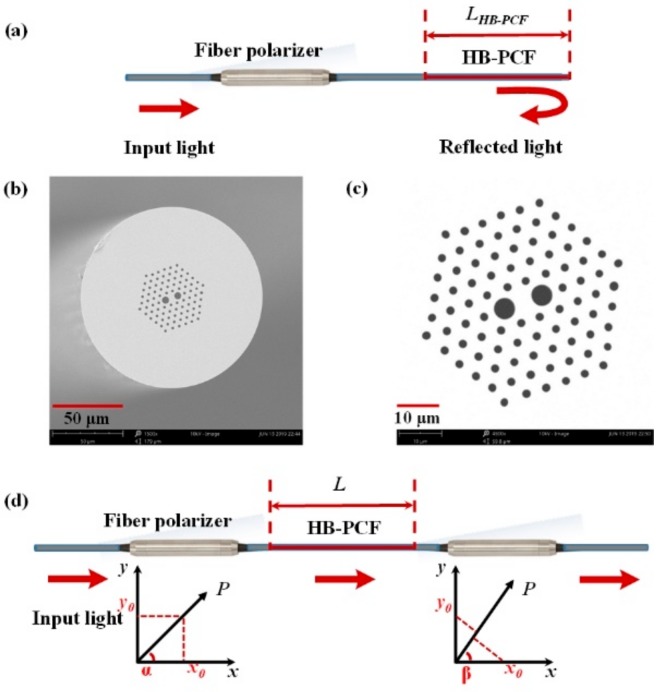
(**a**) Configuration of the proposed reflective Lyot filter. Scanning electron microscope (SEM) micrograph of (**b**) the fiber cross-section. Scale bar: 50 μm. (**c**) the hexagonal air/silica microstructure. Scale bar: 10 μm. (**d**) The equivalent transmissive Lyot filter of the proposed reflective Lyot filter.

Figure 1 is the configuration of the proposed reflective Lyot filter used for gas pressure sensing. Apart from the HB-PCF used as the birefringent medium, the configuration of reflective Lyot filter only uses one fiber polarizer (ILP-1550, LIGHTCOMM, Shenzhen, China), instead of two polarizers in the conventional Lyot filter, which simplifies the structure and decreases the cost. The employed HB-PCF is a commercial polarization-maintaining fiber (PM-125-05, YOFC, Wuhan, China) with a birefringence Δ*n* of 8.73 × 10^−4^. To observe the structure of the HB-PCF, the morphologies of the fiber cross-section and the hexagonal air/silica microstructure are characterized by a scanning electron microscope (SEM), and the obtained micrographs are shown in Figure 1b,c, respectively. From the SEM micrographs, we can see that the core of the employed HB-PCF is roughly an ellipse with a long axis of 7 μm and a short axis of 4.5 μm, which is surrounded by a hexagonal air/silica microstructure cladding that encircled by a pure silica cladding. The diameters of the fiber and the hexagonal air/silica microstructure cladding are 125 μm and 41.7 μm, respectively. The array of the air-holes in the microstructure cladding mainly consists of air-holes with an average diameter of 1.8 μm, except for two air-holes with diameter of 4.72 μm neighboring the fiber core. The birefringence of the PCF is mainly attributed to the geometric birefringence induced by the two large air-holes. In addition, the end of the HB-PCF is well-cleaved, so the fiber end can act as a fiber reflector based on the Fresnel reflection to reflect the input light back into HB-PCF. As the angle between the optic axis of the fiber polarizer and the slow-axis of the HB-PCF is α, the polarization state of the input light is *α*. However, for the reflected light, its polarization state is changed to *β* by the Fresnel reflection. Then, the reflected light goes back into the fiber polarizer.

According to the optical path of the light transmitting in the reflective Lyot filter, the proposed reflective Lyot filter can be equivalent to a transmissive Lyot filter shown in Figure 1d. The equivalent transmissive Lyot filter is formed by sandwiching an HB-PCF-based birefringence cavity (*L* in length) with two fiber polarizer. The polarization directions of the two fiber polarizers are *α* and *β* with respect to the slow-axis of the PM fiber. The working principle of this Lyot filter is that after being linearly polarized by the fiber polarizer, the input light splits up into two co-propagating orthogonally polarized beams along the HB-PCF with a relative phase difference Δ*φ*, then recombining at the fiber polarizer, thereby resulting in the interference.

For the output light of the reflective Lyot filter, the normalized intensity is [22]:

(1) $$I=\sin^{2}\alpha \sin^{2}\beta +\cos^{2}\alpha \cos^{2}\beta +\frac{1}{2}\sin2\alpha \sin2\beta \cos\Delta \varphi$$

Here:

(2) $$\begin{array}{c} \Delta \varphi =\frac{2\pi \cdot \Delta n\cdot L}{\lambda }\;\\ \Delta n=n_{slow}-n_{fast} \end{array}$$

*n_slow_* and *n_fast_* are the refractive indices at the slow and fast axes of the HB-PCF, and *L* is the length of the birefringence cavity. Under the condition of Δ*φ* = *2mπ* or Δ*φ* = *(2m + 1)π* (*m* = 0, 1, 2…), the normalized intensity reaches its maximum or minimum, respectively. The maximum and minimum normalized intensity are:

(3) $$\begin{array}{l} I_{\max}=\sin^{2}\alpha \sin^{2}\beta +\cos^{2}\alpha \cos^{2}\beta +\frac{1}{2}\left|\sin2\alpha \sin2\beta \right|\\ I_{\min}=\sin^{2}\alpha \sin^{2}\beta +\cos^{2}\alpha \cos^{2}\beta -\frac{1}{2}\left|\sin2\alpha \sin2\beta \right| \end{array}$$

According to Equation (3), we can know that the fringe visibility of the interference spectrum is dependent on the angle *α* and *β*, simultaneously. To get a high fringe visibility, the angle *α* and *β* should be set reasonably in the experiment. 

The free spectrum range (FSR) of the interference spectrum is [22]:

(4) $$FSR=\frac{\lambda^{2}}{\Delta n\cdot L}$$

The wavelength of the dip in the interference spectrum is:

(5) $$\lambda_{\min}=\frac{2\cdot \Delta n\cdot L}{2m+1}$$

which indicates that the dip wavelength *λ_min_* is dependent of the fiber birefringence *Δn* and the length of the birefringence cavity *L*. For the HB-PCF-based reflective Lyot filter, when the gas pressure is applied on HB-PCF, the original refractive indices distribution along the orthogonal axes of the HB-PCF is changed in a different amount, then resulting in the variation of the fiber birefringence Δ*n* and bringing about the shift of the dip wavelength *λ_min_* accordingly. In other words, the different variations of the dip wavelengths correspond to the different gas pressures applied on the HB-PCF. Based on this mechanism, we can achieve the gas pressure sensing by simply monitoring the variations of the dip wavelengths in the interference spectrum of the reflective Lyot filter.

## 3. Experiment Research

**Figure 2:**
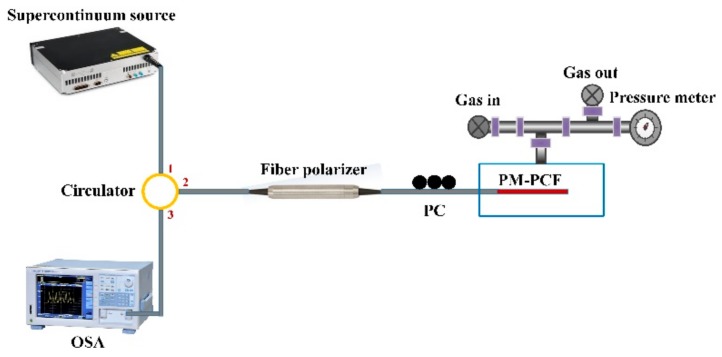
Experiment setup for gas pressure sensing with the reflective Lyot filter. PC: Polarization controller. OSA: Optical spectrum analyzer.

**Figure 3:**
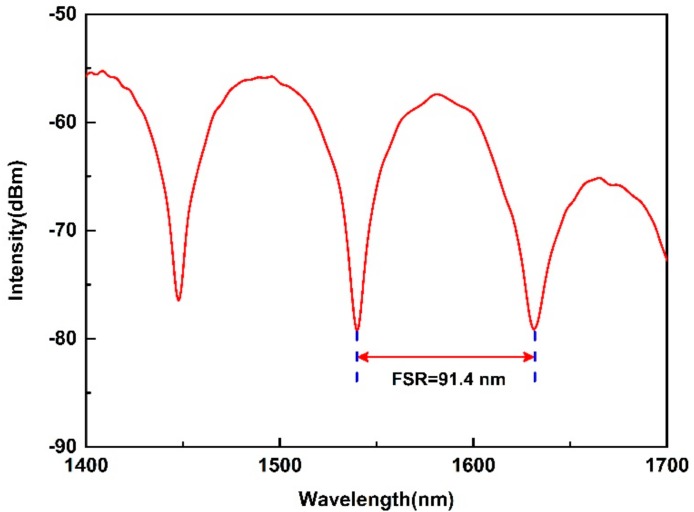
Original interference spectrum of the reflective Lyot filter in the wavelength range from 1400 to 1700 nm.

Figure 2 presents the experiment setup for gas pressure sensing with the reflective Lyot filter. A supercontinuum source (YSL Photonics SC-5) operating in the waveband from 1000 nm to 1700 nm is employed as the input light source, and the proposed reflective Lyot filter is used as the gas pressure sensor in the sensing system. As mentioned above, the fringe visibility of the interference spectrum is dependent on the angle *α* and *β*, simultaneously. In the experiment, we added a polarization controller (PC, FPC562, THORLABS, Newton, NJ, USA) to adjust the angle *α* to get a high fringe visibility. Yet, in view of the fact that a desired fringe visibility can be acquired by reasonably pre-setting the angle *α*, the PC is not essential in the reflective Lyot filter. The length *L* of the employed HB-PCF is about 1.5 cm. In addition to being used as the birefringent medium, the HB-PCF also plays the role of the sensor head in the sensor. The sensor head is sealed in an air chamber, and a commercial gas pressure generator is connected to the air chamber to precisely control the inner gas pressure. Meanwhile, a digital gas pressure meter is employed to measure the actual inner gas pressure. By this method, different gas pressures can be precisely applied on the sensor head. A commercial optical spectrum analyzer (OSA, AQ6370C, YOKOGAWA, Tokyo, Japan) was employed to monitor the output light real-timely. Moreover, the supercontinuum source, the reflective Lyot filter, and the OSA were connected to the ports 1, 2, and 3 of an optical circulator (OC-1550, LIGHTCOMM, Shenzhen, China), respectively. Thus, the input light from the supercontinuum source can enter into the reflective Lyot filter and then be on-line measured by the OSA. The original spectrum of the output light from the proposed reflective Lyot filter is shown in Figure 3, which is a typical interference spectrum in that the intensity changes periodically depending on the wavelength. By adjusting the PC, the obtained fringe visibility in the interference spectrum is about 25 dB, and the FSR near 1550 nm is 91.4 nm, corresponding to the birefringence cavity length *L* of ~3 cm.

**Figure 4:**
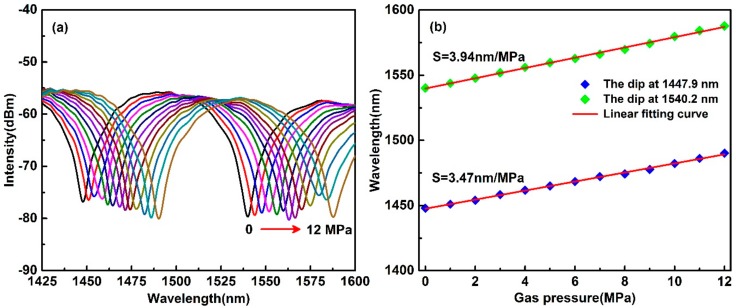
(**a**) Interference spectra evolution of the reflective Lyot filter under the gas pressure ranging from 0 to 12 MPa. (**b**) Relationship between the dip wavelength and the applied gas pressure.

To investigate the gas pressure response of the reflective Lyot filter, the inner gas pressure of the air chamber was precisely adjusted from 0 to 12 MPa with a step of 1 MPa by the commercial gas pressure generator, and the interference spectra of the reflective Lyot filter were recorded at each gas pressure test point after a 10 min interval. Figure 4a is the recorded interference spectra in the waveband from 1425 to 1600 nm. It can be observed that the dip wavelength shifts to the longer wavelength with the increase of applied gas pressure. For the dip at the wavelength of 1447.9 nm and 1540.2 nm, the gas pressure-induced maximum wavelength shifts are 42.2 and 47.6 nm, respectively, and the relationship between the two dip wavelengths and the applied gas pressure in the range from 0 to 12 MPa is shown in Figure 4b. From Figure 4b, one can note that the two dip wavelengths follow the applied gas pressure linearly. By linear fitting, the obtained the slopes of the two linear fitting curves are 3.47 and 3.94, indicating that the achieved gas pressures sensitivities are 3.47 nm/MPa and 3.94 nm/MPa, respectively.

For the proposed reflective Lyot filter, the variation *Δλ_min_* of the dip wavelength induced by the gas pressure is:

(6) $$\Delta \lambda_{\min}=\frac{2\cdot \Delta n\prime \cdot L}{2m+1}$$

Combining with Equation (6), Equation (5) can be simplified as:

(7) $$\Delta \lambda_{\min}=\frac{\Delta n\prime }{\Delta n}\lambda_{\min}$$

where Δ*n′* is the gas pressure-induced birefringence in the HB-PCF. For the HB-PCF, the fiber birefringence is originated from the asymmetry stress distribution induced the two large air-holes near the fiber core. When the gas pressure is applied on the HB-PCF, a compressive deformation in the air/silica microstructure of the HB-PCF is induced by the pressure and then changes the original stress distribution in the HB-PCF. With the change of the stress distribution, the corresponding refractive indices *n_slow_* and *n_fast_* are changed simultaneously. Due to the asymmetry of the fiber structure, the gas pressure-induced refractive index variation between *n_slow_* and *n_fast_* is different, which results in the birefringence variation *Δn′* in HB-PCF. According to Equation (7), we can know that the birefringence variation *Δn′* leads to the shift of the dip wavelength in the interference, which agrees well with the experimental results shown in Figure 4.

The gas pressure sensitivity of the proposed sensor can be derived from Equation (7) as:

(8) $$\begin{array}{l} \frac{d\Delta \lambda_{\min}}{\mathrm{dp}}=\frac{\lambda_{\min}}{\Delta n}\frac{d\Delta n\prime }{\mathrm{dp}}{=K}_{P}\frac{\lambda_{\min}}{\Delta n}\\ {\;K}_{P}=\frac{d\Delta n\prime }{\mathrm{dp}}=\frac{dn_{\mathrm{slow}}}{\mathrm{dp}}-\frac{dn_{\mathrm{fast}}}{\mathrm{dp}} \end{array}$$

where *K_P_* is the birefringence-pressure coefficient of the employed HB-PCF employed in the reflective Lyot filter, which is a constant that only depends on the HB-PCF itself. It can be seen from Equation (8) that the gas pressure sensitivity of the proposed sensor is a constant that only rests with the birefringence-pressure coefficient *K_P_* of the employed HB-PCF. The gas pressure sensitivity can be further improved by employing fibers with higher birefringence-pressure coefficient. 

**Figure 5:**
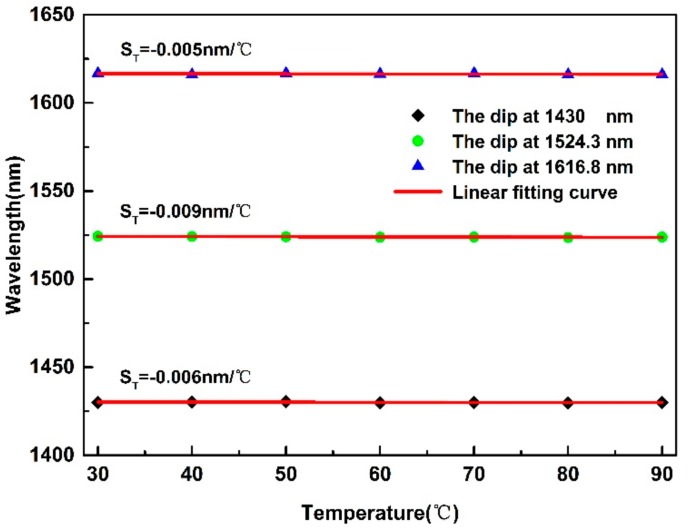
Temperature response of the reflective Lyot filter under the temperature ranging from 30 to 90 °C.

In the practical application, the cross-sensitivity from temperature is a key problem of the gas pressure sensor. To research the temperature cross-sensitivity in the proposed reflective Lyot filter-based gas pressure sensor, we fabricated a new sample and conducted a temperature test. The HB-PCF was heated from 30 °C to 90 °C with a step of 10 °C by a column oven. At each temperature test point, the corresponding interference spectrum was recorded. Figure 5 shows the wavelengths of the selected dips under different temperatures. It can be clearly seen from Figure 5 that the dip wavelengths are almost stable at different temperatures, and the obtained maximum temperature sensitivity is −0.009 nm/°C, corresponding to the temperature cross-sensitivity of −2.28 × 10^−3^ MPa/°C. A comparison of the temperature sensitivities achieved by different birefringent fibers-based sensors is presented in Table 1. From Table 1, we can know that conventional birefringence fibers-based sensors are usually sensitive to temperature [31,32,33]. In comparison, the temperature sensitivities of PCF-based sensors are fairly low. The obtained experimental result indicates that the proposed gas pressure sensor is temperature independent, and the low temperature cross-sensitivity mainly results from the small thermal optical coefficient of birefringence of HB-PCF. As the material along the orthogonal axes of HB-PCF is the same, the temperature-induced refractive index variation along the orthogonal axes is almost the same, which means that the birefringence Δ*n* almost stays the same, so the dip wavelength is insensitive to temperature variation, corresponding to the low temperature cross-sensitivity.

## 4. Conclusions

In conclusion, a temperature-independent gas pressure sensor with high birefringence photonic crystal fiber-based reflective Lyot filter has been proposed and demonstrated in this paper. The reflective Lyot filter simply consists of a fiber polarizer and a segment of HB-PCF. The investigations on the gas pressure-response characteristic and the temperature-response characteristic of the reflective Lyot filter were conducted in experiment, and the obtained experiment results suggest that such a fiber-reflective Lyot filter is sensitive to gas pressure rather than temperature. For the practical application, the proposed gas pressure sensor overcomes the cross-sensitivity between gas pressure and temperature, which is expected to be available for gas pressure monitoring.

## Figures and Tables

**Table 1:** Temperature sensitivity of several birefringent fibers.

Fiber Type	Temperature Sensitivity
Polarization maintaining side-hole fiber [31]	140 pm/°C
PANDA fiber [32]	−1.46 nm/°C
High-birefringence elliptic fiber [33]	−472 pm/°C
Asymmetric two-hole fiber [34]	2.22 nm/°C
Polarization maintaining PCF [35]	4.1 pm/°C
HB-PCF in our work	−9 pm/°C
